# Evaluating scientific theories as predictive models in language neuroscience

**DOI:** 10.1101/2025.08.12.669958

**Published:** 2025-08-12

**Authors:** Chandan Singh, Richard J. Antonello, Sihang Guo, Gavin Mischler, Jianfeng Gao, Nima Mesgarani, Alexander G. Huth

**Affiliations:** 1 Microsoft Research, Redmond, WA, USA; 2 Electrical Engineering Department, Columbia University, NY, USA; 3 Zuckerman Institute, Columbia University, NY, USA; 4 Neuroscience Department, University of California, Berkeley, CA, USA; 5 Statistics Department, University of California, Berkeley, CA, USA

## Abstract

Modern data-driven encoding models are highly effective at predicting brain responses to language stimuli. However, these models struggle to *explain* the underlying phenomena, i.e. what features of the stimulus drive the response? We present Question Answering encoding models, a method for converting qualitative theories of language selectivity into highly accurate, interpretable models of brain responses. QA encoding models annotate a language stimulus by using a large language model to answer yes-no questions corresponding to qualitative theories. A compact QA encoding model that uses only 35 questions outperforms existing baselines at predicting brain responses in both fMRI and ECoG data. The model weights also provide easily interpretable maps of language selectivity across cortex; these maps show quantitative agreement with meta-analyses of the existing literature and selectivity maps identified in a follow-up fMRI experiment. These results demonstrate that LLMs can bridge the widening gap between qualitative scientific theories and data-driven models.

## 1 Introduction

The advent of powerful deep learning models has provided neuroscientists with new tools for modeling how the brain encodes information across language, vision, and audition (1, 2, 3). One such tool is the *language encoding model*, which allows neuroscientists to predict the relationship between a language stimulus and its evoked brain response with unprecedented accuracy (4,5,6,7). However, these increasingly large, black-box models are unable to succinctly *explain* their predictions. This has challenged our notion of what it means to understand a natural phenomenon: if our model of the brain is as inscrutable as the brain itself, does that model advance science? Or must our understanding take the form of a scientific theory that can be expressed in words?

To meet this challenge, we present Question Answering encoding models, a method for evaluating qualitative scientific theories as predictive models. First, theories about language in the brain (e.g. *Some brain areas selectively respond to language about time*) are recast as yes/no questions about a piece of text (e.g. *Does the input mention time?*). An LLM is then used to annotate natural language stimuli from a neuroimaging experiment by answering these questions about each segment of text. We then fit linear models that use these annotations as features to predict brain responses, yielding interpretable, highly predictive encoding models. Using as few as 35 questions, QA encoding models outperformed even state-of-the-art black-box encoding models for both fMRI and ECoG.

Each feature in a QA encoding model corresponds to a single theory. Thus each voxel’s linear model weight for a particular feature is highly interpretable, showing which brain areas’ responses match the corresponding theory. We confirmed the reliability of these maps by showing that they match meta-analyses of the existing literature (8). We further evaluated these maps using follow-up fMRI experiments. Specifically, we used generative causal testing (9), which constructs synthetic stories where each paragraph corresponds to a particular theory. We then measured the average fMRI response to each paragraph and found strong agreement with the QA maps. Our results demonstrate the power of QA encoding models to quantitatively evaluate qualitative scientific theories using large-scale data.

## 2 QA encoding models for fMRI are highly accurate and compact

**Figure 1:**
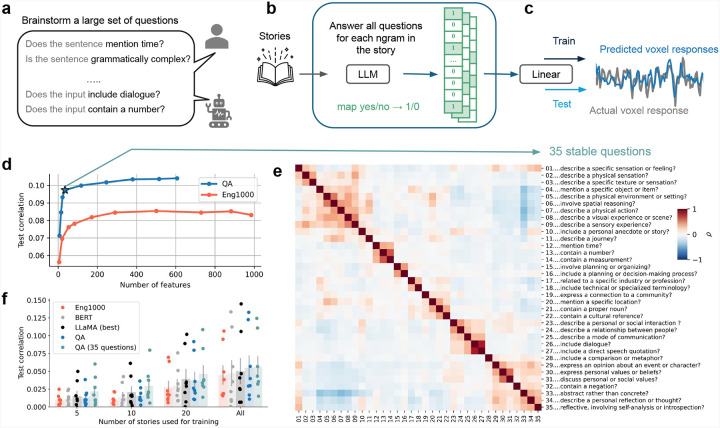
QA encoding models effectively predict fMRI voxel responses from LLM answers to questions. (a) We first enumerated a set of 606 qualitative theories cast as yes/no questions through brainstorming and GPT-4 assistance. (b) 20 hours of narrative stories were converted to 606 interpretable, binary features by having LLMs answer each question for every 10-gram in the stories. (c) These features were then used to build voxelwise encoding models. Voxelwise BOLD responses were recorded using fMRI as human subjects listened to the narrative stories. Regularized linear encoding models were fit to predict each voxel response from the binary features; encoding models were tested by predicting responses on held-out fMRI data. (d) To compress the resulting 606-question encoding models, sparse stability selection was used during fitting. Very few questions were required to achieve strong prediction performance: with only 35 questions, the QA model achieved a test correlation of 0.097, a 13.9% improvement over the best performance (averaged over 3 subjects) attained by the much larger Eng1000 baseline word embedding model. (e) The 35 selected questions and correlations between their answers on the 20 hours of stimuli. They span a variety of topics. (f) Average test encoding performance across cortex for the QA model and baselines on the three original subjects (20 hours of fMRI data each) and 5 additional subjects (5 hours each). The 35-question QA model outperformed the state-of-the-art black-box model (which uses hidden representations from the LLaMA family of LLMs) by 12.0% when trained on all the story data. The model’s compactness yields greater relative improvements in data-limited scenarios; when trained on only 5 stories per subject it outperforms the baseline LLaMA model by 43.3%.

To construct QA encoding models, we began by enumerating a set of 606 plausible neurolinguistic theories expressed as yes/no questions. We generated these questions by first manually writing many examples and then prompting GPT-4 (10) to create more using various strategies, like having GPT-4 list semantic properties of narrative sentences, summarize n-grams from the training data, or generate questions similar to single-voxel explanations found in a prior work (11) (Fig. 1a). We then used an ensemble of LLMs to annotate each 10-gram in 20 hours of narrative stories with the answer to each question (Fig. 1b, see Methods). These answers serve as an interpretable binary-valued text embedding (12). Next, these embeddings were used to fit a linear encoding model for each voxel in 3 human subjects from passive language listening fMRI data (13). The encoding models were then tested by predicting held-out fMRI data and computing the correlation between predictions and actual data (Fig. 1c).

To evaluate which of the 606 questions were useful for predicting brain responses, we employed sparse stability selection (14) of regressors during model fitting (Fig. 1d). This process selected questions that were individually strong predictors (Fig. S1a) and that represented a diverse set of information (Fig. S1b). The QA model that selected only 35 questions (hereafter QA-35) achieved a test correlation of 0.097, already a 13.9% improvement over the best performance attained by Eng1000, a baseline interpretable word embedding model consisting of 985 word-level features (15). We visualized the questions in QA-35 along with the correlation matrix of their answers across the stimulus and found that they span a variety of semantic concepts (Fig. 1e). A majority of the questions are loosely clustered into a handful of high-level categories: questions about *tactile sensations*, *visuospatial information*, *numerical information*, *planning*, *communication*, and *abstract beliefs or values*. As these categories are almost entirely what our model relies upon to make its inferences, they provide additional insight into the large-scale organization principles, such as grounded cognition (16), that govern the structure of cortical language representations (15).

We further evaluated the performance of QA-35 on held-out data for the original 3 subjects as well as 5 additional subjects that were not involved in the question selection. We found that it outperformed even black-box encoding models that use the hidden representations of an advanced LLM, LLaMA (17), by an average of 12.0% when models were trained on all available fMRI data (7) (Fig. 1f). The compactness of QA-35 additionally enables improved data efficiency, leading to larger relative improvements for scarce or low-quality fMRI training data. For example, when models were trained with only 5 stories (roughly 75 minutes of data), the relative improvement over the best baseline was 43.4%.

## 3 Interpreting cortical maps from QA encoding models

The compactness and linearity of QA-35 provide an opportunity to evaluate which areas of cortex are best captured by each qualitative theory. QA-35 predicts the BOLD response timecourse of each voxel as a weighted combination of the answers to 35 questions. Thus, if responses in some voxel are explained by a particular theory, the weight for the corresponding question in that voxel will be high. For example, a voxel that is selective for language about locations should have a high regression weight for the question *Does the input mention a specific location?*

**Figure 2:**
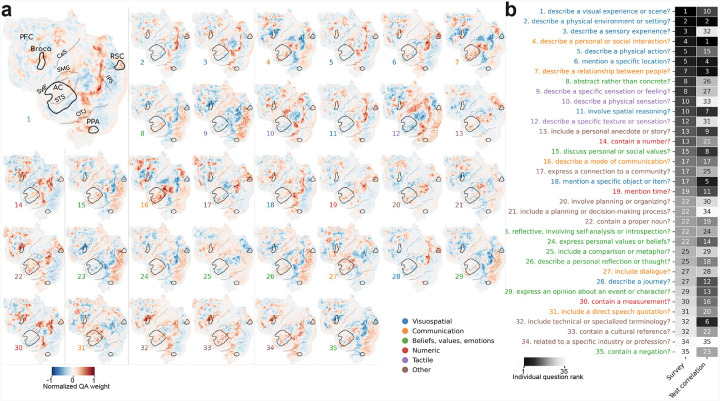
Interpreting cortical maps from QA encoding models. The 35-question QA encoding model in Fig. 1 yields weights that predict each voxel’s responses from each question’s answers; we can interpret these weights as cortical maps of semantic selectivity. (a) These semantic selectivity maps recover known selectivity patterns as well as uncovering new ones. For example, the weights for a question asking about whether the input mentions *locations* (question 6) have high values for regions known to have selectivity for place concepts: retrosplenial cortex (RSC), the parahippocampal place area (PPA), and the occipital place area (OPA). For brevity we show only the left hemisphere in a single subject (S02); see the full-cortex weights for multiple subjects in Section S3.3. Regions annotated are prefrontal cortex (PFC), Broca’s area, central sulcus (CeS), supramarginal gyrus (SMG), the Sylvian fissure (SylF), auditory cortex (AC), superior temporal sulcus (STS), occipitotemporal junction (OTJ), intraparietal sulcus (IPS), retrosplenial cortex (RSC), and parahippocampal place area (PPA). (b) To compare the findings in these selectivity maps against expert opinion, we collected the judgements of the importance of each of the 35 questions in QA-35 for predicting brain responses to language via an anonymous survey. We compared the average ranking of each question based on the survey (1 being the most important) to the ranking of each question based on its individual prediction performance, measured via test correlation. We found a somewhat positive correlation of 0.31 between the two rankings (*p* = 0.071). Many of the top questions ranked by experts were related to visuospatial concepts. Some questions, such as question 32 about *technical or specialized terminology*, are considerably more important for prediction than expert rankings suggested.

Fig. 2a shows the encoding weights across the 35 questions for the left hemisphere of subject S02. The weights show many patterns that are consistent with known neuroscientific principles and theories. For the *locations* question (question 6), the map shows high weights for regions known to have selectivity for place concepts: retrosplenial cortex (RSC), the parahippocampal place area (PPA), and the occipital place area (OPA) (18). Visuospatial information strongly predicts activations in the occipitoparietal junction (19), whereas commmunication and speech information heavily predicts activations in inferior temporal (IT) lobe (9). Information about tactile sensations uniquely activates the supramarginal gyrus, which is home to somatosensory association cortex (8). See full weights for all 35 questions in Fig. S7.

To compare the findings in these 35 selectivity maps against expert opinion, we conducted an anonymous survey asking researchers to provide their judgement of each question’s importance for predicting brain responses to language using a five-point Likert scale (e.g. 1 = “Not at all important”, 5 = “Extremely important”). The survey was sent out to four relevant mailing lists and yielded 12 responses; see full survey details in the Methods. Fig. 2b compares the average survey rating of each question (1 being the most important) to the question’s rank based on its individual prediction performance, measured via test correlation. We found a somewhat significant positive correlation of 0.31 between the two rankings (*p* = 0.071). Many of the top questions ranked by experts were related to visuospatial concepts. A handful of questions, such as *Does the input include technical or specialized terminology?* and *Does the input contain a cultural reference?* were considerably more important for prediction than expected by experts, likely because they are highly abstract and do not readily correspond to well-known theories about the drivers of cortical organization. These questions tended to correspond to complex patterns of activation in frontal lobe, near Broca’s area, as well as along the superior temporal sulcus. See full survey results in Fig. S6.

## 4 Quantitatively evaluating cortical maps from QA encoding models

**Figure 3:**
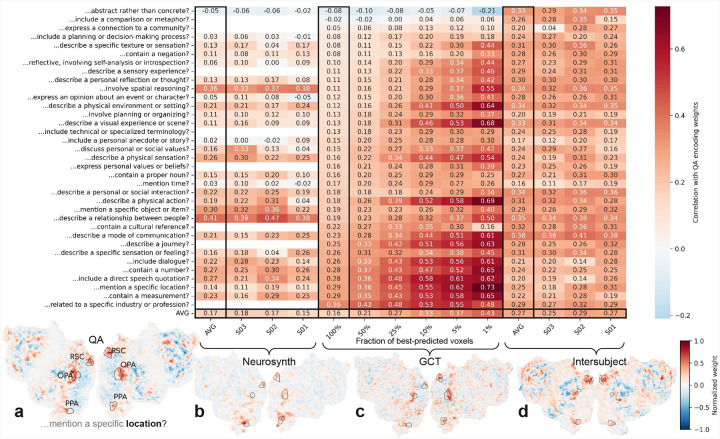
Quantitatively evaluating QA cortical selectivity maps corresponding to 35 stable questions. We evaluate the weights from the 35-question QA encoding model in Fig. 1 as cortical maps of semantic selectivity. (a) As a running example, we show the weights for a question asking about *locations* in subject S02 that displays selectivity for well-studied location-selective regions, such as RSC, OPA, and PPA. (b) To quantitatively evaluate whether the QA weights match known findings in the literature, we compared the QA weights against cortical maps from Neurosynth, a meta-analysis of the neuroscience literature that computed selectivity maps by testing the voxel-level association between keywords in paper abstracts with cortical maps reported in the paper. We found that 27 out of 35 questions from our model had a reasonable match to a keyword available in Neurosynth (see Table S1), but we note that matches are often imprecise. We computed the correlations between the QA weights and the Neurosynth maps (mapped to subject coordinates, masked to the 10% best-predicted voxels by the QA model) and found reasonable agreement: 26 of the 27 correlations were positive and the mean correlation of 0.17 was statistically significant (*p* < 10^−5^, permutation test). (c) To validate the potentially new findings in the QA weights, we ran a follow-up experiment using generative causal testing (GCT). Specifically, we constructed synthetic stimuli designed to test each individual question and then measured the average response to these synthetic stimuli for subject S02. We computed the Pearson correlation between each GCT map and the QA weight and found that the mean correlation of 0.16 was significant (*p* < 10^−6^, permutation test). Masking the maps to include only well-predicted voxels improves the correlation with the GCT map; 8 out of 35 questions showed a statistically significant correlation for the top 1% of best-predicted voxels (*p* < 0.05, permutation test, FDR-corrected). (d) Finally, we assessed consistency across subjects by evaluating the correlation of the weights for each subject with the mean of the other two (averaged in MNI space then projected to the subject coordinates). The mean correlation of 0.30 was significant (*p* < 10^−6^, permutation test), and each individual correlation was also significant (*p* < 0.05, permutation test, FDR-corrected). The bottom row shows cortical selectivity map for visual comparison: (b) *place* projected onto S02, (c) GCT map for *location* in S02, and (d) QA weights for *location* in S03.

To quantitatively evaluate the interpretation of QA-35 weights as cortical selectivity maps, we performed three robustness analyses. As a running example, we show the weights for the question *Does the input mention a specific location?*; see weights for subject S02 in Fig. 3a.

We first evaluated whether the QA-35 weights matched known selectivity maps from the literature. We compared the QA-35 weights with cortical maps from Neurosynth (8), a meta-analysis of the fMRI literature. Neurosynth used an automated pipeline that tagged voxel coordinates reported in neuroscience papers with keywords from the paper abstract, and then computed a selectivity map using an association test between each voxel and each keyword (see example in Fig. 3b). We manually searched through the keywords in Neurosynth and found a plausible match for 27 out of the 35 questions in QA-35 (see matches in Table S1). We note that matches were often imprecise—e.g. matches for *time* may relate to tasks related to *timing* rather than stimuli associated with the concept of *time*—biasing the correlation between the Neurosynth maps and QA-35 weights downwards. We then compared the Neurosynth maps with their corresponding QA-35 weights for each of three subjects by mapping the Neurosynth maps to subject coordinates, masking them to the 10% of best-predicted voxels by QA-35, and computing the correlation across voxels. The mean correlation of 0.17 across questions was significantly greater than zero (*p* < 10^−5^, permutation test), and 26 of the 27 individual correlations were positive, suggesting broad-scale agreement between the automated maps produced by QA-35 and the aggregated findings contained in Neurosynth.

We further assessed the QA-35 selectivity maps using targeted follow-up experiments. We conducted an fMRI experiment designed to measure selectivity for the underlying 35 questions using generative causal testing (GCT) (9). Specifically, we created two hours of synthetic natural stories, with each story paragraph designed to emphasize an individual question from QA-35. We then measured the average response to the paragraphs for each question in subject S02 and then computed the Pearson correlation between this average response and the QA-35 weights for that question (Fig. 3c). The whole-cortex correlation for 33 of the 35 questions was greater than zero and the mean correlation of 0.16 was significant (*p* < 10^−6^, permutation test). Well-predicted voxels showed better consistency with the GCT scores: the mean correlation when computed only over the best-predicted 1% of voxels (943 voxels) was 0.43, with 8 out of 35 questions yielding significant correlations (*p* < 0.05, permutation test with Benjamini-Hochberg false discovery rate correction (20)). To minimize the amount of new fMRI data to be collected, we reused fMRI responses to GCT synthetic stimuli for the subject S02 collected in a previous work (9), when there was a reasonable match between the QA question and the GCT synthetic stimuli (see Table S2).

Finally, we evaluated the consistency of the QA-35 weights across subjects (see example map for subject S03 in Fig. 3d). Specifically, for each subject we projected the weights for the other two subjects into MNI coordinates, averaged them, projected them to the original subject’s coordinates, and then measured the correlation with the original subject’s weights. The mean correlation of 0.30 was significantly greater than zero (*p* < 10^−6^, permutation test), as was each individual correlation (*p* < 0.05, permutation test, FDR-corrected). The questions with the highest inter-subject correlation often involved aspects of communication (e.g. *communication*, a *relationship*, a *social interaction*) or visual concepts (e.g. describe a *physical environment* or a *visual experience*).

These results demonstrate that the selectivity maps generated by QA-35 reflect known cortical function from the literature, are replicable in new stimuli, and are consistent across subjects.

## 5 QA encoding models for ECoG data

QA encoding models are highly effective at capturing fMRI responses to language, and here we investigate whether this success extends to another neuroimaging modality: electrocorticography (ECoG). We analyzed ECoG data from the Podcasts dataset (21), which contains responses from a total of 1,268 electrodes across nine subjects who each listened to the same 30 minute podcast. As was done with the fMRI data, we generated interpretable features for the stimuli using the questions in QA-35. As ECoG provides higher temporal resolution and sensitivity than fMRI, we generated the features at three contextual timescales: at the word level, with 1.5 seconds of context, and with 3 seconds of context. This yielded a total of 105 lexical and contextual QA features that we used to build our encoding model. We supplemented these QA features with low-level spectral features to capture both low-level acoustic and high-level semantic properties of the stimulus while remaining fully interpretable. From these concatenated features we built a model to predict ECoG responses from the high gamma frequency band at various time lags from word onset (22) (see Methods).

**Figure 4:**
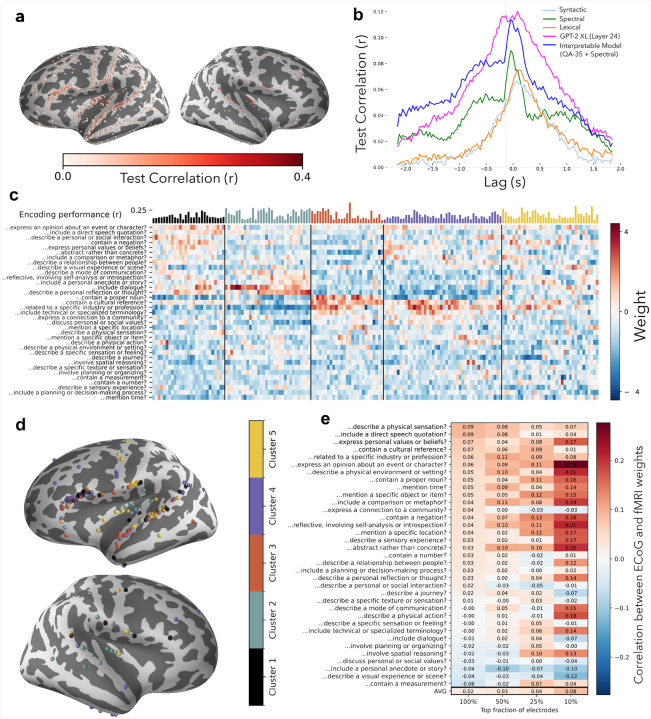
QA encoding models for ECoG data. To evaluate the applicability of QA encoding models to a different neuroimaging modality, we fit encoding models to ECoG responses in 1,268 total electrodes from 9 subjects listening to a 30-minute podcast. (a) For each electrode, we generated an interpretable encoding model by concatenating our 35-question QA encoding model from Fig. 1 with spectral features. We break down our model’s encoding performance, measured at the best performing overall lag, using use a joint template across all subjects. The best-predicted electrodes were found in left STG and near Broca’s area. (b) This model outperforms other interpretable encoding models and achieves similar performance to a non-interpretable baseline model from prior work, which uses GPT-2 XL hidden states as features (*pink*). (c) To understand selectivity trends across these electrodes, we select 166 well-predicted electrodes (see Methods) and visualize the encoding weights for each electrode. These electrodes form 5 high-level clusters with similar selectivity profiles. (d) We visualize these clusters and find that they display moderate spatial homogeneity, despite being aggregated across subjects. (e) We further investigate spatial selectivity by computing the spatial correlation of ECoG electrode weights with fMRI voxel weights for each question. We find statistically significant correlations when selecting different fractions of the best-predicted electrodes; a paired t-test between fMRI-ECoG correlations and permuted fMRI-ECoG correlations yields *p* = (8.3 × 10^−4^, 3.6 × 10^−4^, 3.4 × 10^−5^, 2.7 × 10^−5^) for top electrode fractions (100%, 50%, 25%,10%)

Fig. 4a shows the encoding performance of the resulting QA-35+Spectral model at the best performing lag for all 1,268 electrodes across all 9 subjects on a template brain. Many electrodes show strong performance with the QA features. The best prediction performance was found in left STG and near Broca’s area, with similar performance in the right hemisphere analogues of these regions.

We compared the interpretable QA-35+Spectral model with various baseline encoding models from prior work on this dataset (21). We selected 166 electrodes based on their minimum prediction performance across all encoding models (see Methods) and performed the remaining analyses on the selected electrodes. Fig. 4b shows the performance of our interpretable model and baselines for each lag relative to word onset, averaged across the thresholded electrodes. As baselines, we built a model that only uses the spectral features, a syntactic model that includes simple part-of-speech / dependency parsing information, a black-box lexical embedding space (*en.core.web.lg*) and a black-box contextual embedding space from a large language model (*GPT*-*2 XL*, Layer 24) that was used in prior work (21). Our interpretable model achieved prediction performance that roughly matched the black-box LLM baseline at many lags and outperformed the other models, in spite of the fact that these questions were originally chosen for their ability to predict temporally coarser fMRI data. As all of the feature spaces contain information about the immediate temporal context, we were often able to predict neural response before word onset (23,24).

To identify patterns of selectivity across electrodes, we visualized each electrode’s feature weights across the questions from QA-35 (Fig. 4c). Because the ECoG models included three separate features for each question that correspond to the three timescales used, we averaged weights across the timescales. Hierarchical clustering of these weights across electrodes illustrates various clusters of electrodes whose responses are driven by different types of questions. These clusters show some anatomical separation, as illustrated in Fig. 4d. For example, cluster 2, which strongly loads on the question *Does the input include dialogue?*, is largely located near primary auditory cortex. In contrast, cluster 3, which has high loadings on the *…contain a proper noun?* and *…contain a cultural reference?* questions, is spread throughout superior temporal gyrus and inferior frontal gyrus. This demonstrates the potential for these QA models, whose 35 features were not optimized for intracranial encoding, to reveal new patterns of response tuning.

Finally, to further test the consistency of our approach, we investigated the degree to which anatomical patterns across the two modalities aligned. Fig. 4e shows the cross-modality correlations between the fMRI weights and the ECoG weights for each question. All fMRI and ECoG weights were first mapped to the same FreeSurfer fsaverage template coordinates (25). fMRI weights were mapped from volumetric coordinates to vertices on the template, and then a distance-weighted average of the nearest 10 vertices was used as a proxy for the fMRI weight for each electrode location. The correlation between these interpolated weights from fMRI and ECoG was then directly computed. Overall, the correlation between fMRI and ECoG maps is significantly greater than zero (*p* = 8.3 × 10^−4^; paired t-test between the correlations of the fMRI and ECoG maps with and without permutation), with stronger correlation when only the best-performing electrodes are considered. The few negatively correlated features, such as *…describe a journey?* or *…describe a visual experience or scene?*, likely reflect undersampling of the relevant cortical areas in the ECoG dataset. The significant correlations between the spatial patterns uncovered by fMRI and ECoG QA encoding models demonstrate that the uncovered patterns are robust across datasets and modality.

## 6 Discussion

Many prior studies have demonstrated the predictive power of black-box encoding models in language neuroscience across modalities such as fMRI, ECoG, EEG, and MEG (1,4,5,6,7). However, these models have been criticized for their inability to produce accurate and reliable explanations (26,27) and attempts to explicitly interpret these models have been limited (28, 29, 30). Our work addresses this criticism by directly constructing a fully interpretable encoding model using LLM-generated annotations.

The methods here are related to work that has used LLMs as tools for data annotation (31) in domains such as medicine (32,33), finance (34), and general natural-language processing (35,36). They also share a looser connection with interpretable text representations, which are often built by averaging word-level embeddings (15, 37, 38) or ngram-level embeddings (39, 40). In contrast, we use LLMs as tools not just for annotating and representing data, but for formulating and testing qualitative theories.

This distinction provides an important scientific benefit: it enables empirical evaluation of theory’s predictive power and iteration on theories to refine their accuracy and detail. The process of generating these theories may be aided by methods for the post-hoc interpretation of black-box models (41,42,11) that can generate refined descriptions of encoding model behavior in specific regions or domains of interest. QA encoding models can efficiently evaluate the predictive power of these theories and guide the design of follow-up experiments that causally test these theories, e.g. through generative causal testing (9).

While effective, QA encoding models have two major limitations. First, QA encoding models are computationally intensive, requiring an LLM call for every question at every timepoint. This is often prohibitively expensive, but will likely become more feasible as LLM inference costs continue to decrease. Additionally, a single LLM model can be distilled to answer multiple questions simultaneously when the questions are predefined (36, 12). Second, QA encoding models rely on the underlying LLM’s ability to faithfully answer the given yes-no questions (see evaluation in the Methods). If an LLM is unable to accurately answer the questions, it compromises the interpretability of the QA encoding models. Thus, QA encoding models require the use of strong LLMs that can accurately answer the set of chosen questions. This challenge will also likely diminish as LLM capabilities improve.

The work here opens many avenues for future research. One approach can explore using QA encoding models for various input modalities. Modern multimodal foundation models can answer textual questions about diverse modalities, including audio and images (43), enabling annotation for a broader range of stimuli. Additionally, QA encoding models can be readily applied to emerging scanning modalities (e.g. 7T laminar fMRI or NeuroPixels), which may reveal selectivity patterns that were previously undetectable. Finally, while we take an initial step towards characterizing cortex-wide selectivity map, QA encoding models can easily be tailored to answer more targeted theories about selectivity in the brain. In the appendix we include example vignettes illustrating this application–e.g. using QA encoding models to identify monosemantic voxels (Section S2.1), describe distinctive characteristics of the language network (Section S2.2), and decoding the answers to different questions (Section S2.3).

QA encoding models offer a general method for evaluating theories through data-driven predictions. In this paper, we demonstrate how modern LLMs can enhance our understanding of cognitive brain processes. Through simple prompt-based methods, we annotate large natural datasets to recover selectivity theories that traditionally required decades of painstaking controlled experiments and pave the way for many similar future discoveries. More broadly, the core concept of QA encoding models–using foundation models to operationalize qualitative theories about the natural world–has applications well beyond neuroscience. QA encoding models will help advance scientific discourse toward increasingly nuanced, data-supported explanations.

## Supplementary Material

1 Materials and Methods
Supplementary Text
Figures S1 to S10
References 44–56
Tables S1 to S3

## Data Availability

All newly collected fMRI data will be made publicly available upon acceptance. Data for fitting encoding models and generating explanations is available on OpenNeuro: openneuro.org/datasets/ds003020 (fMRI) and openneuro.org/datasets/ds005574/versions/1.0.2 (ECoG). Code for running all experiments (as well as applying QA encoding models in new settings) is available on Github at github.com/microsoft/automated-brain-explanations. Code uses python 3.10, huggingface transformers 4.29.088–100, sklearn 1.2.040, and OpenAI API GPT-4 (*gpt*-*4*-*0125*-*preview*).
