# Supplementary material for: Evaluating scientific theories as predictive models in language neuroscience
Source: bioRxiv. 2025 Aug 12:2025.08.12.669958. Preprint. [Version 1] doi: 10.1101/2025.08.12.669958 (PMC12363820; doi:10.1101/2025.08.12.669958)
Supplement: 1 — Materials and Methods Supplementary Text Figures S1 to S10 References 44–56 Tables S1 to S3 [file NIHPP2025.08.12.669958V1-supplement-1.pdf]

bioRxiv preprint doi: https://doi.org/10.1101/2025.08.12.669958; this version posted August 12, 2025. The copyright holder for this preprint (which was not certified by peer review) is the author/funder, who has granted bioRxiv a license to display the preprint in perpetuity. It is made available under aCC-BY 4.0 International license.

Supplementary materials for "Evaluating scientific theories as predictive models in language neuroscience"

Chandan Singh[1,*,†] Richard J. Antonello[2,3,†] Sihang Guo[4] Gavin Mischler[2,3] Jianfeng Gao[1]
Nima Mesgarani[2,3,‡] Alexander G. Huth[4,5,‡]

[1]Microsoft Research, Redmond, WA, USA
[2]Electrical Engineering Department, Columbia University, NY, USA
[3]Zuckerman Institute, Columbia University, NY, USA
[4]Neuroscience Department, University of California, Berkeley, CA, USA
[4]Statistics Department, University of California, Berkeley, CA, USA

*Corresponding author. Email: chansingh@microsoft.com
†These authors contributed equally to this work.
‡ These authors contributed equally as senior authors of this work.

This PDF file includes:

Materials and Methods
Supplementary Text
Figures S1 to S10
References 44-56
Tables S1 to S3

bioRxiv preprint doi: https://doi.org/10.1101/2025.08.12.669958; this version posted August 12, 2025. The copyright holder for this preprint (which was not certified by peer review) is the author/funder, who has granted bioRxiv a license to display the preprint in perpetuity. It is made available under aCC-BY 4.0 International license.

S1 Materials and methods

fMRI data collection This study uses two MRI datasets: one for fitting encoding models and a second for evaluating cortical selectivity maps. The original set is described and made publicly available in previous work (*13*, *44*), while details of the newly collected second dataset are described here. Functional magnetic resonance imaging (fMRI) data were collected from a single subject (S02) in the original set as stories were visually presented at approximately conversational cadence. We collected two hours of data corresponding to 9 stories. Participants passively listened to the stories without making any responses. All subjects were healthy and had normal hearing. The experimental protocol was approved by the Institutional Review Board at the University of Texas at Austin and written informed consent was obtained.

All MRI data were collected on a 3T Siemens Skyra scanner at the University of Texas at Austin using a 64-channel Siemens volume coil. Functional scans were collected using a gradient echo EPI sequence with repetition time (TR) = 2.00 s, echo time (TE) = 30.8 ms, flip angle = 71°, multi-band factor (simultaneous multi-slice) = 2, voxel size = 2.6mm x 2.6mm x 2.6mm (slice thickness = 2.6mm), matrix size = 84x84, and field of view = 220 mm. Anatomical data were collected using a T1-weighted multi-echo MP-RAGE sequence with voxel size = 1mm x 1mm x 1mm following the Freesurfer morphometry protocol (*25*).

fMRI data preprocessing All functional data were motion corrected using the FMRIB Linear Image Registration Tool (FLIRT) from FSL 5.0. FLIRT was used to align all data to a template that was made from the average across the first functional run in the first story session for each subject. These automatic alignments were manually checked for accuracy.

Low frequency voxel response drift was identified using a 2nd order Savitzky-Golay filter with a 120 second window and then subtracted from the signal. To avoid onset artifacts and poor detrending performance near each end of the scan, responses were trimmed by removing 20 seconds (10 volumes) at the beginning and end of each scan. This process eliminated the 10-second silent period and the first and last 10 seconds of each story. The mean response for each voxel was subtracted and the remaining response was scaled to have unit variance.

Generating questions for QA encoding models To generate the questions underlying QA encoding models, we prompted GPT-4 (*45*) (*gpt-4-0125-preview*) with 6 prompts that aimed to elicit semantic information that was useful for predicting fMRI responses (precise prompts in Section S3.1). This included directly asking the LLM to use its knowledge of neuroscience, to brainstorm semantic properties of narrative sentences, to summarize examples from the input data, and to generate questions similar to single-voxel explanations found in a prior work (*11*). Many of the prompts included examples of diverse, reasonable questions, incorporating the author's domain knowledge. After deduplication, this process yielded 606 questions (see all the questions on Github).

Extracting QA features For answering questions, we took the mean of the answers from Mistral-7B (*46*) (*mistralai/Mistral-7B-Instruct-v0.2*), LLaMA-3 8B (*47*) (*meta-llama/Meta-Llama-3-8B-Instruct*) with two prompts, and GPT-4 (*gpt-4-0125-preview*). QA features were extracted using 64 AMD MI210 GPUs, each with 64 gigabytes of memory. See all prompts in Section S3.1.

If an LLM is unable to accurately answer the questions, this compromises the interpretability of the QA encoding models. Thus, QA encoding models require the use of high-performing LLMs, and the set of chosen questions must be accurately answered by these models. Section S3.4 provides an analysis of the question-answering accuracy of different LLMs and finds that the LLMs used in this study can reliably answer the queried questions.

Regression modeling Each subject's fMRI data consists of approximately 100,000 voxels; we pre-processed it by running principal component analysis (PCA) and extracting the coefficients of the top 100

bioRxiv preprint doi: https://doi.org/10.1101/2025.08.12.669958; this version posted August 12, 2025. The copyright holder for this preprint (which was not certified by peer review) is the author/funder, who has granted bioRxiv a license to display the preprint in perpetuity. It is made available under aCC-BY 4.0 International license.

components for each TR. We then fit ridge regression models to predict these 100 coefficients. We still evaluated the models in the original voxel space (by applying the inverse PCA mapping and measuring the correlation between the response and prediction for each voxel in the test set). To handle temporal sampling, we followed the approach in prior works (*15*, *1*); an embedding was computed at the timepoint where each word occurred in the input story, and these embeddings were interpolated using Lanczos resampling. Embeddings at each timepoint were computed from the ngram consisting of the 10 preceding words. We selected the best-performing hyperparameters via cross-validation on 5 time-stratified bootstrap samples of the training set. The best ridge parameters were chosen from 12 logarithmically spaced values between 10 and 10,000. To model temporal delays in the fMRI signal, we selected between adding 4, 8, or 12 time-lagged duplicates of the stimulus features. After fitting and evaluating the encoding model, we averaged across the temporal delay dimension of the weights for visualization and comparison (e.g. Fig. 3).

Selecting stable QA features We selected a compact feature set using stability selection (*14*). Specifically, we fit multi-task Elastic net with 10 logarithmically spaced regularization parameters ranging from 10^{-3} to 1 using the *MultiTaskElasticNet* class from scikit-learn (*48*). We randomly sampled the training dataset by 50% five times and fit the Elastic net model to each of the sets. We then selected only the only the stable features for each regularization parameter, i.e. those that were consistently chosen across all five training sets for a given regularization parameter. After feature selection, we refit the Ridge regression model using only the selected features.

Baselines We compared QA encoding models to Eng1000, an interpretable baseline developed in the neuroscience literature specifically for the task of predicting fMRI responses from narrative stories (*15*). Each element in an Eng1000 embedding corresponds to a co-occurence statistic with a different word, allowing full interpretation of the underlying representation in terms of related words. We additionally compared QA encoding models to embeddings from BERT (*49*) (*bert-base-uncased*) and LLaMA models (*17*,*47*). For each subject, we swept over 5 layers from LLaMA-2 7B (*meta-llama/Llama-2-7b-hf*, layers 6, 12, 18, 24, 30), LLaMA-2 70B (*meta-llama/Llama-2-70b-hf*, layers 12, 24, 36, 48, 60), and LLaMA-3 8B (*meta-llama/Meta-Llama-3-8B*, layers 6, 12, 18, 24, 30). We selected the best layer using cross-validation and then reported its test performance.

Significance testing of cortical map correlations In Section 4, we performed permutation tests to compare the correlation between different cortical maps. All permutation tests were performed relative to a null distribution of 2,000 randomly selected response TRs from the training data for each subject. Since these responses were recorded during a passive listening task, they generally represent semantic processing information present in the stimulus while preserving correlation structure among the voxels.

Expert survey To evaluate the findings in the QA selectivity maps against expert opinion, we conducted an anonymous survey asking researchers to provide their judgment of each question's importance for predicting brain responses to language using a five-point Likert scale (e.g. 1 = "Not at all important", 3 = "Moderately important", 5 = "Extremely important"). We included the 35 questions in QA-35. From the remain 571 questions, we additionally included 5 of the 15 most highly predictive questions and 5 of the 15 most poorly predictive questions, manually selected to be diverse (see selected questions in Fig. S1(c)). The exact survey prompt was "Below, we have listed 45 potential properties of language stimuli. For each property, we would like you to rate how important that property is for predicting brain responses to language. Specifically, for each property, please rate the degree to which knowing that a stimuli possesses that property would be useful for you to determine which brain regions might be responsive to that stimuli."

The survey was sent out to four relevant mailing lists (cvnet: *cvnet-request@lawton.ewind.com*, visionscience: *visionlist@visionscience.com*, and computational neuroscience: *comp-neuro@neuroinf.org*,

bioRxiv preprint doi: https://doi.org/10.1101/2025.08.12.669958; this version posted August 12, 2025. The copyright holder for this preprint (which was not certified by peer review) is the author/funder, who has granted bioRxiv a license to display the preprint in perpetuity. It is made available under aCC-BY 4.0 International license.

comp-neuro@neuroinformatics.be) and yielded 12 responses. The survey protocol was approved by the Institutional Review Board at Microsoft Research and written informed consent was obtained.

ECoG data details The Podcast ECoG dataset (*21*) comprises electrocorticography (ECoG) recordings from nine epilepsy patients who listened to a 30-minute audio podcast. We summarize the dataset and its preprocessing details here (further details can be found in the dataset paper). The 1,330 electrodes in the initial dataset are filtered down to 1,268 electrodes by removing electrodes with poor localization or noisy power spectrum density. All electrode visualizations were plotted on the *fsaverage* template. We utilized the high-gamma band power of the signal for all of our analyses, extracted by applying a Butterworth band-pass infinite impulse response filter from 70Hz to 200Hz.

ECoG modeling details We predicted ECoG responses from the high gamma frequency band at various time offsets from word onset following a prior work (*22*). Specifically, for each of 128 equally-spaced lags in between -2 and 2 second offsets from word onset, we predicted the electrode response as a function of the QA features. Ridge regression was used to produce a linear model from the stimulus features to the responses for each (electrode, lag) pair. Since the amount of training data is small, model performance was estimated using 5-fold cross-validation of the dataset, training on 80% of the data and testing on the remaining 20% for each of 5 folds. Within each fold, the regularization parameter α of this regression was estimated using boostrapped cross-validation.

For follow-up analysis, we selected electrodes only if they had a best-lag encoding performance of at least $r = 0.06$ across all examined feature spaces (*syntactic*, *spectral*, *En.core.web.lg*, *GPT-2 XL*, *Interpretable*). This performance threshold was selected using the elbow method (see Fig. S10). The selection process yielded 166 electrodes.

bioRxiv preprint doi: https://doi.org/10.1101/2025.08.12.669958; this version posted August 12, 2025. The copyright holder for this preprint (which was not certified by peer review) is the author/funder, who has granted bioRxiv a license to display the preprint in perpetuity. It is made available under aCC-BY 4.0 International license.

S2 Additional analyses using QA encoding models

S2.1 Evaluating single-question QA encoding models

(a) (b)

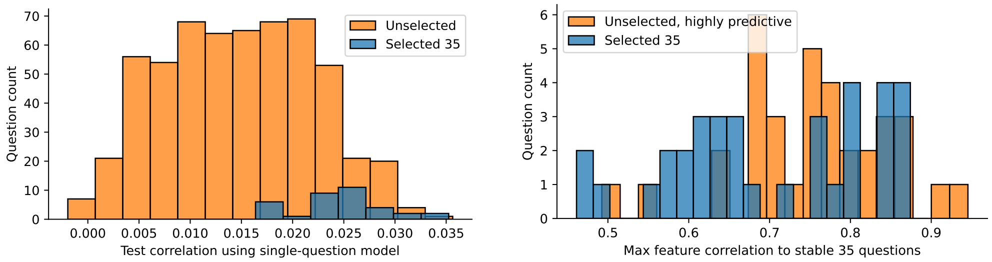

(c)

Question	Mean test correlation
...contain words with strong visual imagery?*	0.036
...part of a narrative?	0.033
...describe an act of communication or interaction with others?	0.030
...describe an interpersonal misunderstanding or dispute?*	0.030
...describe an emotional response to a specific event?*	0.030
...mention of a physical object or item?	0.030
...involve a social or interpersonal interaction?	0.030
...involve the description of an emotional response?	0.030
...mention of a scientific fact or concept?	0.030
...describe a an emotional reaction to a surprise or unexpected event?	0.030
...use irony or sarcasm?*	0.029
...contain a first-person narrative?	0.029
...contain a description of an interaction that led to a misunderstanding or conflict?	0.029
...first-person pronoun in the input?*	0.029
...include a recounting of an impactful or emotional dialogue?	0.029
...part of a legal document or text?*	0.002
...mention a fitness or sports achievement?	0.002
...religious or spiritual practice mentioned?	0.002
...describe a scientific experiment or discovery?*	0.002
...referencing legal matters or rights?	0.002
...involve a financial planning or investment strategy?	0.002
...discuss a breakthrough in medical research?*	0.001
...mention of a war, battle, or military event?	0.001
...a well-known quote or saying?	0.000
...involve a coding or programming concept?*	0.000
...discussion about politics or government?	-0.000
...discuss a natural disaster or emergency situation?	-0.001
...educational lesson or class described?*	-0.001
...reference a religious or spiritual concept?	-0.002
...mention of a natural disaster?	-0.002

Figure S1: Single-question QA encoding models. (a) We computed the prediction performance (mean test correlation) when building QA encoding models using the features extracted by only a single question. The single-question models corresponding to the 35 selected questions yielded stronger performance (mean correlation of 0.025) than the remaining 571 questions (mean correlation of 0.015). (b) The unselected but highly predictive questions often were highly related to one of the 35 selected questions. To demonstrate this, we computed the maximum correlation between the features extracted for a question and the features extracted for each of the 35 selected questions (excluding the question itself). For the 35 unselected questions with the highest individual predictive performance (orange), the mean maximum correlation was 0.70. This is higher than the mean maximum correlation for the 35 selected questions (blue), which was 0.51. (c) We show the 15 most predictive single questions that were not selected in QA-35 as well as the 15 least predictive single questions. Questions marked with an asterisk were used as part of the external expert survey described in the Methods.

bioRxiv preprint doi: https://doi.org/10.1101/2025.08.12.669958; this version posted August 12, 2025. The copyright holder for this preprint (which was not certified by peer review) is the author/funder, who has granted bioRxiv a license to display the preprint in perpetuity. It is made available under aCC-BY 4.0 International license.

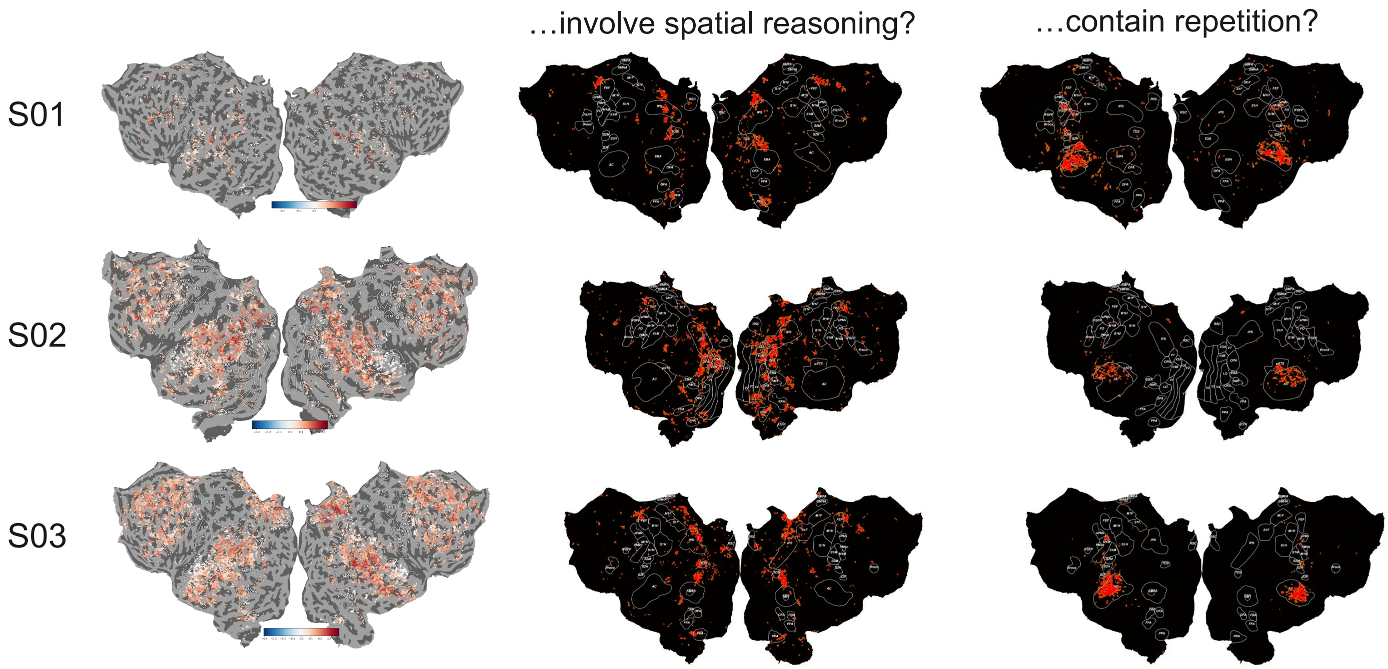

Figure S2: **Monosemanticity in fMRI voxels**. We fit monosemantic encoding models to each voxel by selecting the single-question encoding model that achieved the best cross-validation performance on the training set. The left column displays the difference between the test correlation for the full 35-question model and a monosemantic model. We find that individual questions can predict a small fraction of voxels reasonably well, but most voxels are polysemantic–i.e. incorporating more QA features improves performance. The well-predicted monosemantic regions (white) often correspond to one of two questions: either *involving spatial reasoning* (middle column) or *containing repetition* (right column).

bioRxiv preprint doi: https://doi.org/10.1101/2025.08.12.669958; this version posted August 12, 2025. The copyright holder for this preprint (which was not certified by peer review) is the author/funder, who has granted bioRxiv a license to display the preprint in perpetuity. It is made available under aCC-BY 4.0 International license.

S2.2 Characterizing language network voxels with QA encoding models

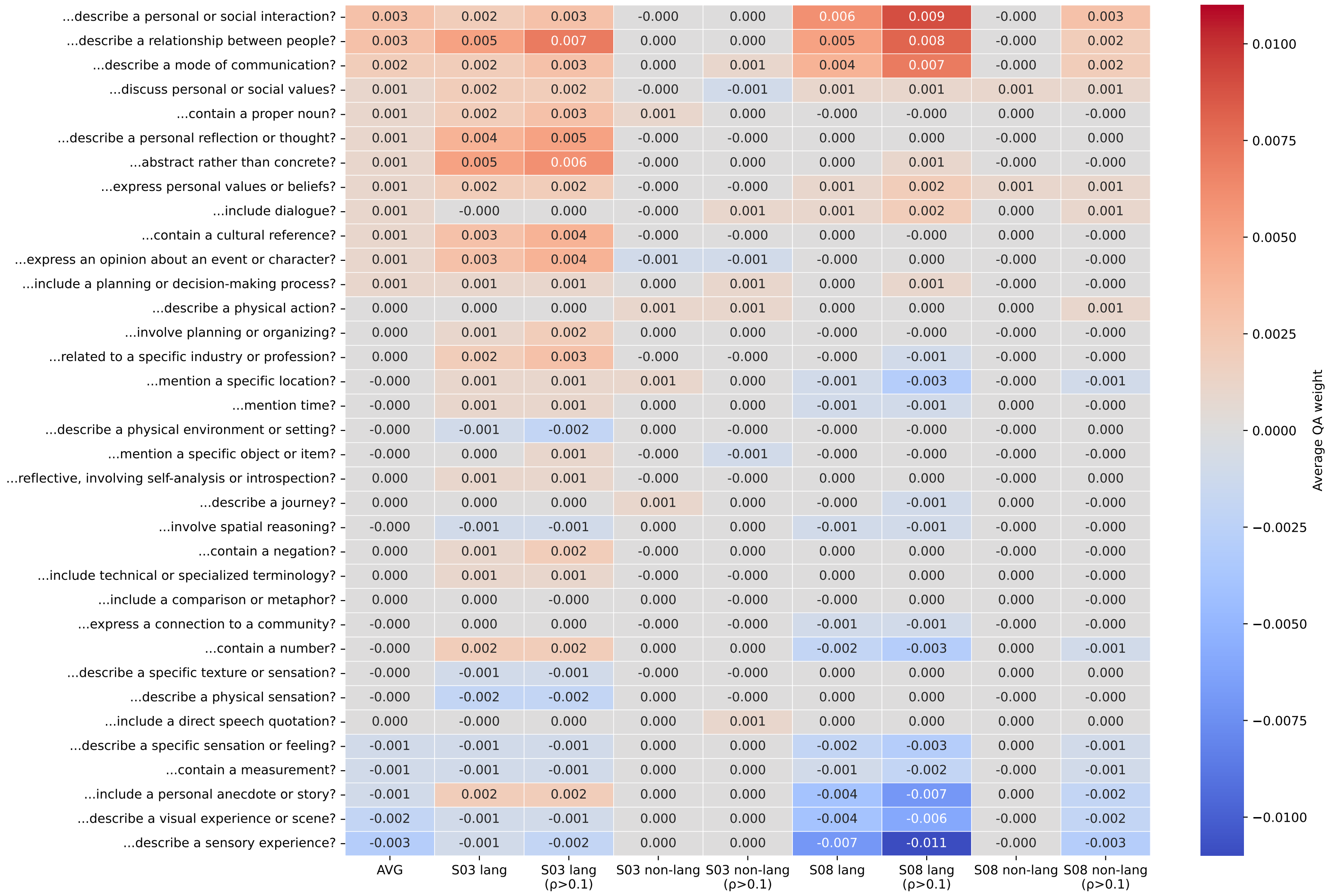

Figure S3: **Characterizing voxels in the language network**. Language network voxels–i.e., voxels that respond significantly more to sentences than non-words–were identified using an auditory language localizer (*50*) task. These voxels were identified as any cortical voxel that passed a one-sided t-test with a significance threshold of $p <= 0.001$ (uncorrected). We then evaluated the overall selectivity of the language network by computing the average QA weight for language-network voxels compared to non-language-network voxels. We further filtered voxels based on whether they were well-predicted by the QA-35, meaning they achieved a test correlation greater than 0.1. Across two subjects, a handful of questions showed distinct differences between language-network and non-language-network voxels.

S2.3 Decoding QA features

While the main results focused so far on encoding models, QA features can instead be used as targets for semantic decoding models, i.e. models that predict the QA answer at a particular timepoint from the recorded brain responses. These decoding models may be useful in different scenarios than encoding models, such as improving brain-machine interfaces (*51*, *52*).

To build decoding models in the fMRI data, we first constructed binary QA labels for each TR by temporally downsampling the binary answers for each 10-gram and thresholding the result at a z-score of 1. We then predicted this label independently for each question using logistic regression. As inputs to

bioRxiv preprint doi: https://doi.org/10.1101/2025.08.12.669958; this version posted August 12, 2025. The copyright holder for this preprint (which was not certified by peer review) is the author/funder, who has granted bioRxiv a license to display the preprint in perpetuity. It is made available under aCC-BY 4.0 International license.

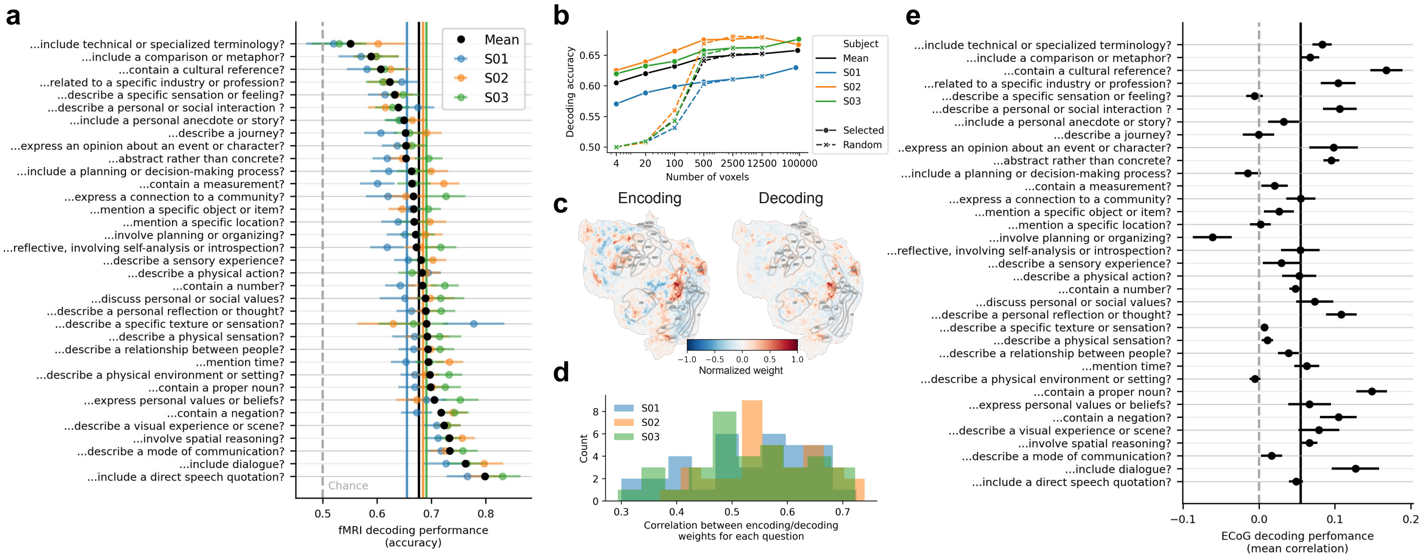

Figure S4: **Decoding individual QA features from fMRI.** (a) We linearly decoded the answers to 35 stable questions for each TR from the four subsequent TRs. Decoding accuracy for all questions was above chance (chance was set at 0.5 by undersampling each question to have 50% yes answers). The three best-decoded questions involve speech and communication. (b) We then decoded the answers using only the responses from voxels that had the largest positive initial decoding weights. This masking strategy yielded fairly high decoding accuracy compared to decoding from randomly selected voxels, and even achieved reasonably high decoding accuracy using only 4 voxels. (c) We then compared the weights for encoding and decoding. Example weights for a question about *locations* are shown for subject S02's left hemisphere, both again emphasizing well-known location-selective regions such as RSC, PPA, and OPA. (d) The weights for encoding and decoding are generally quite similar. The similarity (averaged over the three subjects) is statistically significant for each question ($p < 0.05$, permutation test, FDR-corrected). (e) We performed an analogous decoding analysis using ECoG data and decoded the responses to each question at a much finer timescale. Most questions were decoded above chance, measured using the mean correlation of the decoded label and averaged over 9 subjects. All error bars show the standard error of the mean.

the logistic regression, we used the voxel responses recorded in the four TRs following the decoded TR. To make comparisons clearer, we subsampled the data for each question to equally balance the labels in both the training and testing set.

Fig. S4a shows that all questions were decoded above chance for the three subjects, with a mean decoding accuracy of 0.677. The best-decoded questions often involved communication, e.g. a *speech quotation*, *dialogue*, or a *mode of communication*. To test whether decoding could be performed from a restricted set of voxels, we repeated the decoding experiment using only the responses from voxels that had the largest positive decoding weights. We found that this masking strategy achieved fairly high decoding accuracy compared to decoding from randomly selected voxels (Fig. S4b), and even achieved reasonably high decoding accuracy using as little as 4 voxels (mean decoding accuracy 60.5%).

We then compared the QA weights for encoding and decoding the same question. Interpreting weights from a decoding model can be difficult: even if a concept is reflected in a voxel, it may not be uniquely reflected in the voxel and therefore assigned a low decoding weight (*53*, *54*). Nevertheless, we observed strong alignment between the encoding and decoding weights for each question (see example selectivity for the question *Does the input mention a specific location?* in Fig. S4c). The mean Pearson correlation between encoding and decoding weights for each question was 0.545 (significant with $p <$

bioRxiv preprint doi: https://doi.org/10.1101/2025.08.12.669958; this version posted August 12, 2025. The copyright holder for this preprint (which was not certified by peer review) is the author/funder, who has granted bioRxiv a license to display the preprint in perpetuity. It is made available under aCC-BY 4.0 International license.

10^{-6}, permutation test) and the average correlation across 3 subjects was significant for every question ($p <$ 0.05, permutation test, FDR-corrected). We found that large decoding weights were often concentrated in auditory cortex, more so than encoding weights (see averages in Fig. S5).

We similarly decoded the labels for each question in the ECoG dataset at each timepoint (see Methods). We were again able to decode most questions above chance (Fig. S4e). Taken together, these decoding results further demonstrate that the sparse 35 questions accurately capture major dimensions of semantic and cognitive selectivity on cortex.

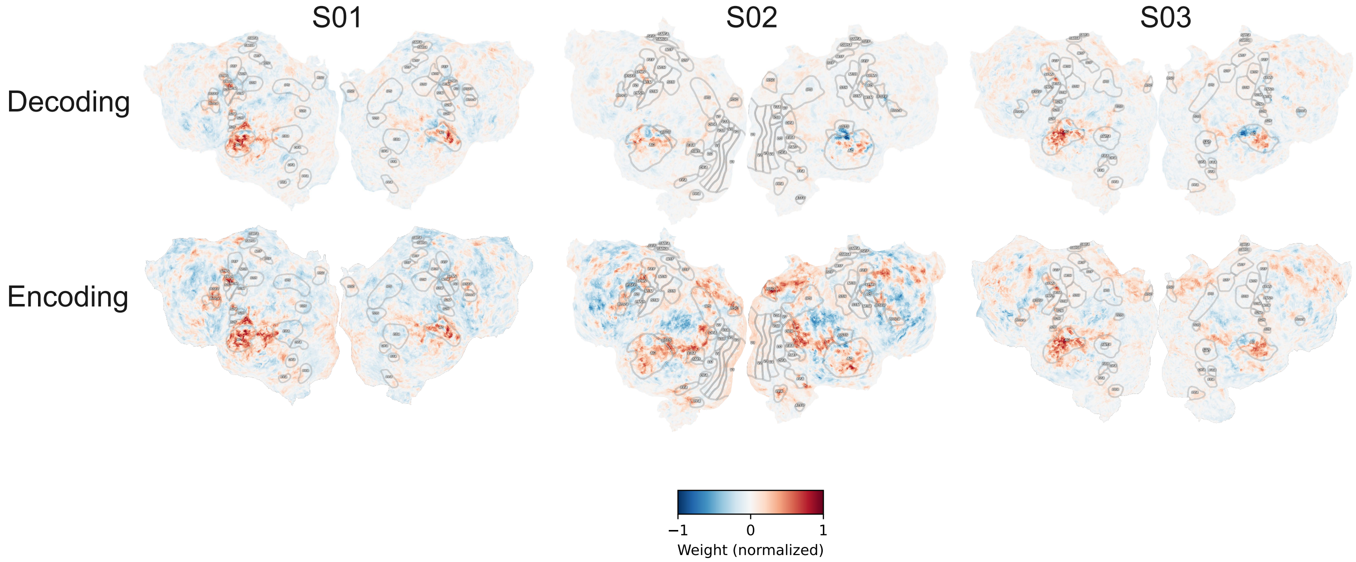

Figure S5: Average decoding and encoding weights across questions. Auditory cortex tends to have the largest values, especially for decoding. This is likely because auditory cortex captures information regarding the local word rate and the QA features are often zero when there are no (or few) words spoken in a TR, a consistent trend across all questions.

bioRxiv preprint doi: https://doi.org/10.1101/2025.08.12.669958; this version posted August 12, 2025. The copyright holder for this preprint (which was not certified by peer review) is the author/funder, who has granted bioRxiv a license to display the preprint in perpetuity. It is made available under aCC-BY 4.0 International license.

S2.4 Extended survey results

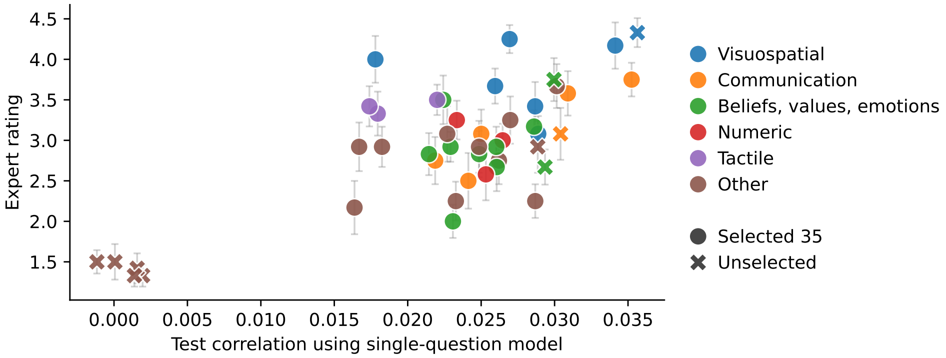

Figure S6: To evaluate the findings of the QA-35 selectivity maps against expert opinion, we conducted an anonymous survey asking researchers to rate questions based on how important they are for predicting brain responses to language using a five-point Likert scale (1 = "Not at all important", 5 = "Extremely important"). We included the 35 questions in QA-35 along with 5 highly predictive and 5 poorly predictive questions from the remaining 571 questions. The survey was sent out to four relevant mailing lists and yielded 12 responses; see full survey details in the Methods. The experts clearly identified the 5 poorly predictive questions (mean rating 1.14) and were moderately successful at ranking the remaining questions (mean correlation 0.37, even after excluding the 5 worst-predicting questions). The raters show mild but significant inter-rater agreement with a Fleiss' kappa of 0.098 ($p < 10^{-3}$, permutation test).

S3 Extended data and methods details

S3.1 Prompting details

See the general prompts used for eliciting questions below. Note that {{*examples*}} was filled in with 5-10 examples representing diverse concepts, e.g. *Does the input contain a number?*, *Does the input mention laughter?*, *Is hair or clothing mentioned in the input?*. See the exact examples in the Github repo.

Question generation prompt 1 *Generate a bulleted list of 500 diverse, non-overlapping questions that can be used to classify an input based on its semantic properties. Phrase the questions in diverse ways.*

Here are some example questions:
{{*examples*}}
Return only a bulleted list of questions and nothing else

Question generation prompt 2 *Generate a bulleted list of 100 diverse, non-overlapping questions that can be used to classify sentences from a first-person story. Phrase the questions in diverse ways.*

Here are some example questions:
{{*examples*}}
Return only a bulleted list of questions and nothing else

Question generation prompt 3 *Generate a bulleted list of 200 diverse, non-overlapping questions that can be used to classify sentences from a first-person story. Phrase the questions in diverse ways.*

bioRxiv preprint doi: https://doi.org/10.1101/2025.08.12.669958; this version posted August 12, 2025. The copyright holder for this preprint (which was not certified by peer review) is the author/funder, who has granted bioRxiv a license to display the preprint in perpetuity. It is made available under aCC-BY 4.0 International license.

Here are some example questions:
{{examples}}
Return only a bulleted list of questions and nothing else

Question generation prompt 4 *Based on what you know from the neuroscience and psychology literature, generate a bulleted list of 100 diverse, non-overlapping yes/no questions that ask about properties of a sentence that might be important for predicting brain activity.*

Return only a bulleted list of questions and nothing else

Question generation prompt 5 *# Example narrative sentences*
{{example sentences from dataset}}

# Example yes/no questions
{{example questions already asked}}

Generate a bulleted list of 100 specific, non-overlapping yes/no questions that ask about aspects of the example narrative sentences that are important for classifying them. Focus on the given narrative sentences and form questions that combine shared properties from multiple sentences above. Do not repeat information in the example questions that are already given above. Instead, generate complementary questions that are not covered by the example questions. Return only a bulleted list of questions and nothing else.

Question generation prompt 6 *Generate more diverse questions that may occur for a single sentence in a first-person narrative story*

Question answering standard prompt

```
<User>: Input text: \{example\}\n
Question: \{question\}\n
Answer with yes or no, then give an explanation.}
```

Question answering few-shot prompt

```
<System>: You are a concise, helpful assistant.\n
<User>: Input text: and i just kept on laughing because it was so
Question: Does the input mention laughter?
Answer with Yes or No.
<Assistant>: Yes
<User> Input text: what a crazy day things just kept on happening
Question: Is the sentence related to food preparation?
Answer with Yes or No.
<Assistant>: No
<User> Input text: i felt like a fly on the wall just waiting for
Question: Does the text use a metaphor or figurative language?
Answer with Yes or No.
<Assistant>: Yes
<User> Input text: he takes too long in there getting the pans from
Question: Is there a reference to sports?
Answer with Yes or No.
```

bioRxiv preprint doi: https://doi.org/10.1101/2025.08.12.669958; this version posted August 12, 2025. The copyright holder for this preprint (which was not certified by peer review) is the author/funder, who has granted bioRxiv a license to display the preprint in perpetuity. It is made available under aCC-BY 4.0 International license.

```
<Assistant>: No
<User> Input text: was silent and lovely and there was no sound except
Question: Is the sentence expressing confusion or uncertainty?
Answer with Yes or No.
<Assistant>: No
<User> Input text: \{example\}
Question: \{question\}Answer with Yes or No.
<Assistant>:
```

S3.2 Cortex map matching details

Table S1: Matches between the questions in QA-35 and Neurosynth keywords.

Question	Neurosynth keyword
...contain a measurement?	arithmetic
...contain a number?	arithmetic
...describe a specific texture or sensation?	sensation
...involve planning or organizing?	planning
...contain a negation?	negative
...contain a proper noun?	nouns
...describe a personal or social interaction ?	social-interaction
...describe a personal reflection or thought?	thoughts
...describe a physical action?	action
...describe a physical sensation?	touch
...describe a relationship between people?	social
...describe a specific sensation or feeling?	sensation
...describe a visual experience or scene?	visual-information
...express an opinion about an event or character?	judgments
...include a direct speech quotation?	communication
...include a personal anecdote or story?	personal
...include dialogue?	communication
...describe a physical environment or setting?	location
...discuss personal or social values?	social
...involve spatial reasoning?	spatial
...mention a specific object or item?	object
...mention a specific location?	place
...describe a mode of communication?	communication
...include a planning or decision-making process?	planning
...abstract rather than concrete?	abstract
...reflective, involving self-analysis or introspection?	thoughts
...mention time?	time-task
...related to a specific industry or profession?	NO MATCH
...express personal values or beliefs?	NO MATCH
...describe a sensory experience?	NO MATCH
...include technical or specialized terminology?	NO MATCH
...contain a cultural reference?	NO MATCH
...include a comparison or metaphor?	NO MATCH
...express a connection to a community?	NO MATCH
...describe a journey?	NO MATCH

bioRxiv preprint doi: https://doi.org/10.1101/2025.08.12.669958; this version posted August 12, 2025. The copyright holder for this preprint (which was not certified by peer review) is the author/funder, who has granted bioRxiv a license to display the preprint in perpetuity. It is made available under aCC-BY 4.0 International license.

Table S2: **Imprecise matches for generative causal testing experiments.** We used generative causal testing to evaluate the questions underlying the fitted QA encoding models. While we built precise matches for 31 of the 35 questions, 4 questions have imprecise matches as they were matched with stimuli from the original GCT study (*9*) rather than constructed specifically for the study here.

Question	GCT driving explanation (imprecise)
Does the sentence involve the mention of a specific object or item?	Descriptive elements of scenes or objects
Does the input include a comparison or metaphor?	abstract descriptions
Does the sentence express a sense of belonging or connection to a place or community?	Relationships
Does the text describe a journey	Spatial positioning and directions

S3.3 Selectivity maps

bioRxiv preprint doi: https://doi.org/10.1101/2025.08.12.669958; this version posted August 12, 2025. The copyright holder for this preprint (which was not certified by peer review) is the author/funder, who has granted bioRxiv a license to display the preprint in perpetuity. It is made available under aCC-BY 4.0 International license.

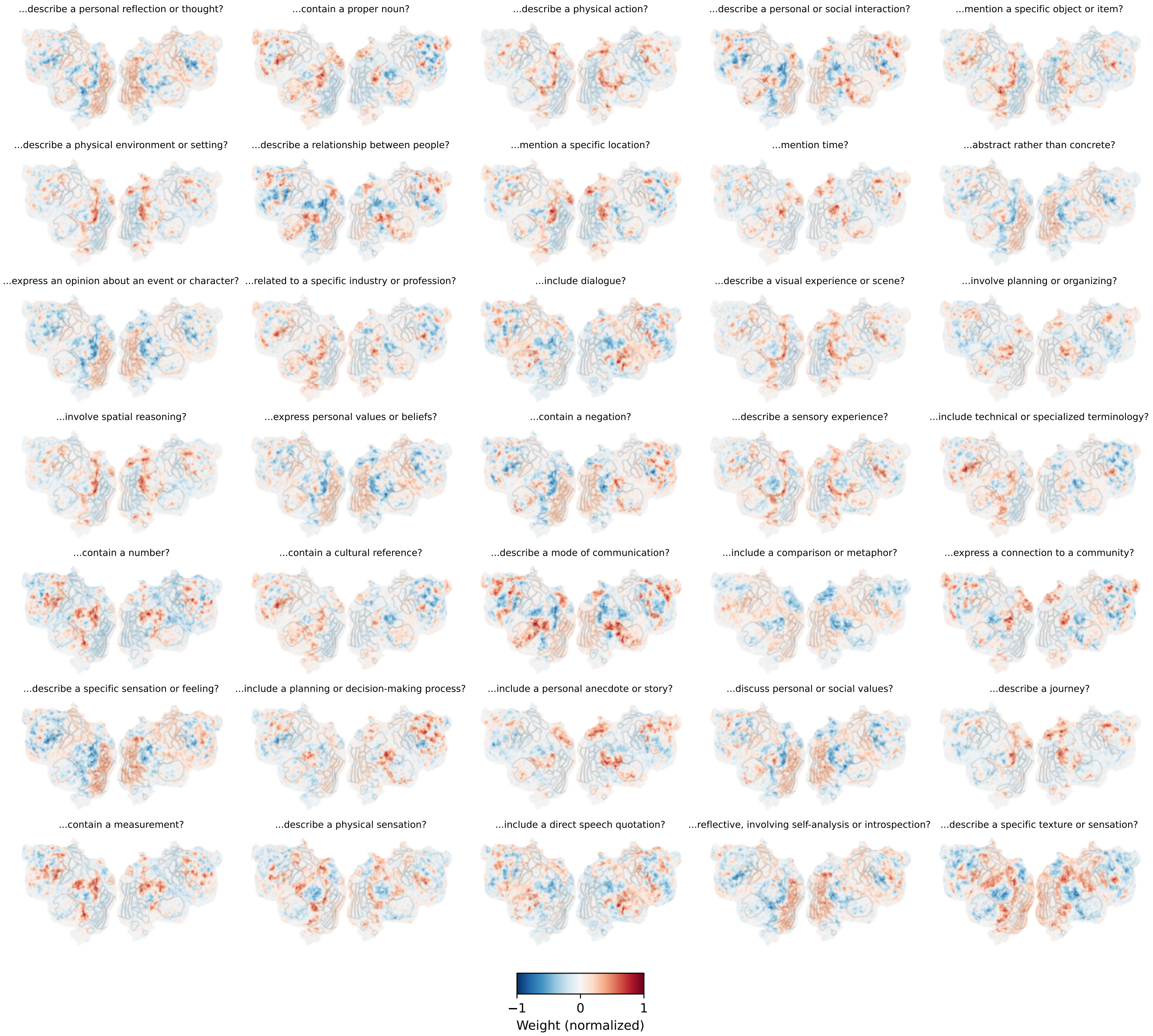

Figure S7: QA encoding weights for each question in a single subject (S02).

bioRxiv preprint doi: https://doi.org/10.1101/2025.08.12.669958; this version posted August 12, 2025. The copyright holder for this preprint (which was not certified by peer review) is the author/funder, who has granted bioRxiv a license to display the preprint in perpetuity. It is made available under aCC-BY 4.0 International license.

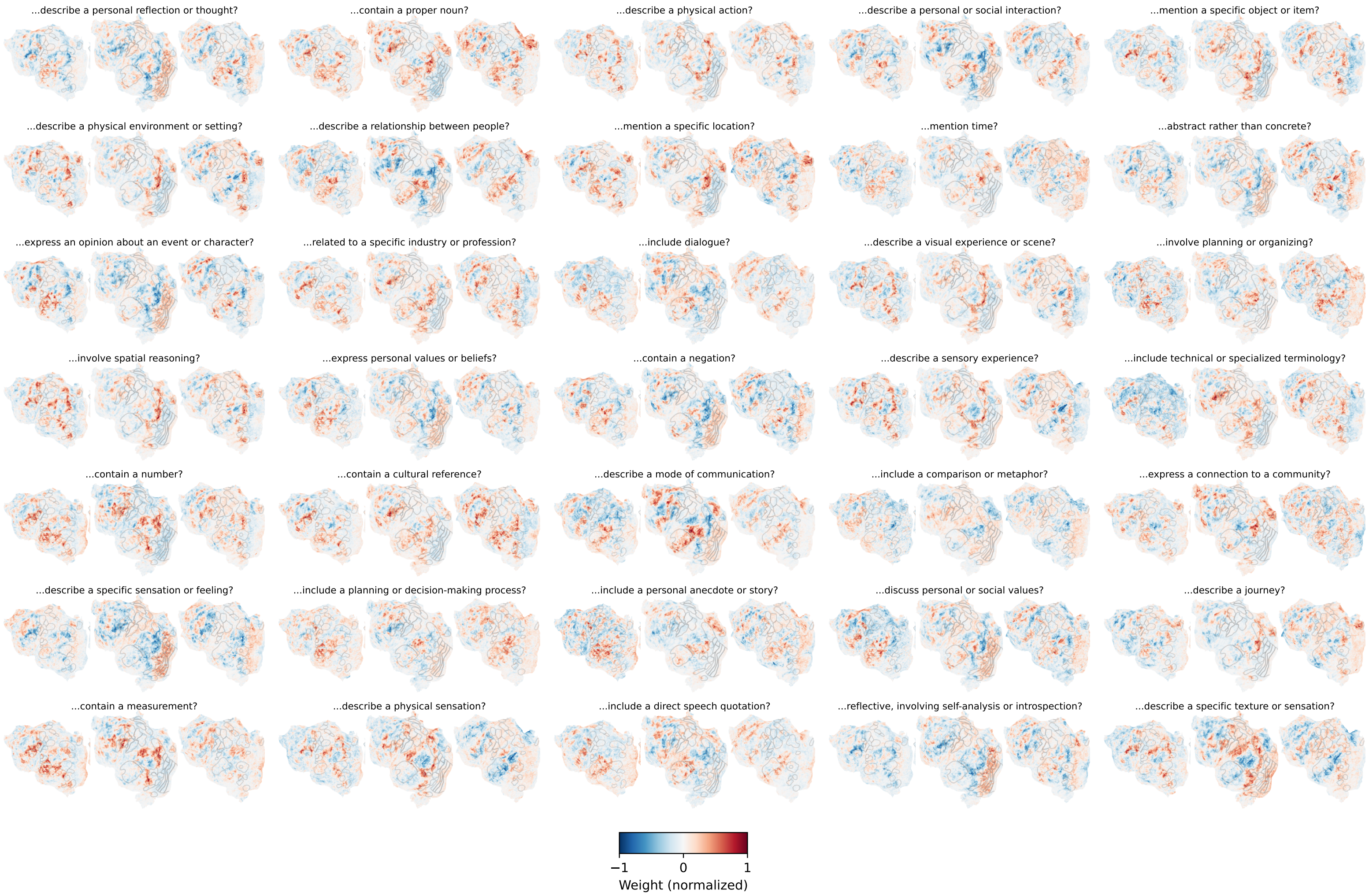

Figure S8: QA encoding weights for each question in QA-35 in left hemisphere for three subjects (S01, S02, and S03 from left to right).

bioRxiv preprint doi: https://doi.org/10.1101/2025.08.12.669958; this version posted August 12, 2025. The copyright holder for this preprint (which was not certified by peer review) is the author/funder, who has granted bioRxiv a license to display the preprint in perpetuity. It is made available under aCC-BY 4.0 International license.

S3.4 Evaluating question-answering faithfulness

	Correlation
Human 1 ⇔ GPT-4	0.94 ± 0.03
Human 2 ⇔ GPT-4	0.91 ± 0.03
Human 1 ⇔ Human 2	0.95 ± 0.02

(a)

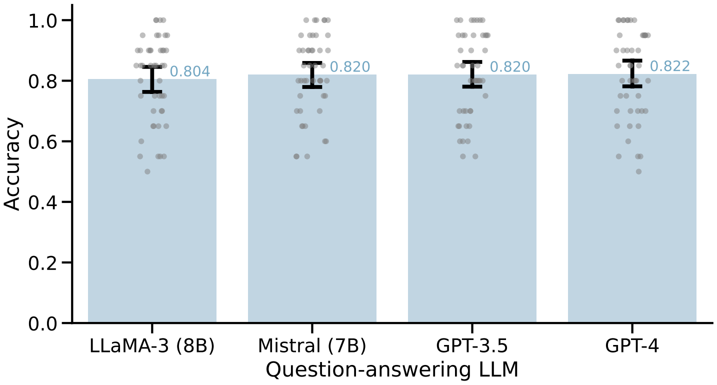

(b)

Figure S9: **Evaluating the question-answering performance of underlying LLMs**. (a) For each of the 35 stable questions, we selected 150 10-grams from the training set that had balanced answers (i.e. 100 yes answers and 100 no answers), resulting in 5,250 (question, 10-gram) pairs. We then had 2 humans manually annotate the answer for each pair. We found that, averaged across questions, the agreement (Pearson correlation) between the human annotations and GPT-4 annotations was comparable to the agreement across the human annotations. Error bars show standard error of the mean. (b) We evaluated the faithfulness of our question-answering models on a recent diverse collection of 54 binary classification datasets (*55*) (see data details in Table S3). These datasets are difficult, as they are intended to encompass a wider-ranging and more realistic list of questions than traditional NLP datasets. Each point shows an individual dataset and error bars show the 95% confidence interval. Fig. S9 shows the classification accuracy for the 3 LLMs used in our methods along with GPT-3.5 (`gpt-3.5-turbo-0125`). On average, each of the LLMs answered these questions with fairly high accuracy, with GPT-4 slightly outperforming the other models. However, we observe poor performance on some tasks, which we attribute to the task difficulty and the lack of task-specific prompt engineering. For example, the dataset yielding the lowest accuracy asks the question *Is the input about math research?*. While this may seem like a fairly simple question for an LLM to answer, the examples in the negative class consist of texts from other quantitative fields (e.g. chemistry) that usually contain numbers, math notation, and statistical analysis. Thus the LLMs answered *yes* to most examples and achieve accuracy near chance (50%). Note that these tasks are more difficult than the relatively simple questions we answer in the fMRI experiments, especially since the fMRI input lengths are each 10 words, whereas the input lengths for these datasets are over 50 words on average (with some inputs spanning over 1,000 words).

S3.5 ECoG electrode selection

bioRxiv preprint doi: https://doi.org/10.1101/2025.08.12.669958; this version posted August 12, 2025. The copyright holder for this preprint (which was not certified by peer review) is the author/funder, who has granted bioRxiv a license to display the preprint in perpetuity. It is made available under aCC-BY 4.0 International license.

Table S3: 54 binary classification datasets along with their underlying yes/no question and corpus statistics from a recent collection (*55*).

Dataset name	Dataset topic	Underlying yes/no question	Examples	Unique unigrams
0-irony	sarcasm	contains irony	590	3897
1-objective	unbiased	is a more objective description of what happened	739	5628
2-subjective	subjective	contains subjective opinion	757	5769
3-god	religious	believes in god	164	1455
4-atheism	atheistic	is against religion	172	1472
5-evacuate	evacuation	involves a need for people to evacuate	2670	16505
6-terorrism	terrorism	describes a situation that involves terrorism	2640	16608
7-crime	crime	involves crime	2621	16333
8-shelter	shelter	describes a situation where people need shelter	2620	16347
9-food	hunger	is related to food security	2642	16276
10-infrastructure	infrastructure	is related to infrastructure	2664	16548
11-regime change	regime change	describes a regime change	2670	16382
12-medical	health	is related to a medical situation	2675	16223
13-water	water	involves a situation where people need clean water	2619	16135
14-search	rescue	involves a search/rescue situation	2628	16131
15-utility	utility	expresses need for utility, energy or sanitation	2640	16249
16-hillary	Hillary	is against Hillary	224	1693
17-hillary	Hillary	supports hillary	218	1675
18-offensive	derogatory	contains offensive content	652	6109
19-offensive	toxic	insult women or immigrants	2188	11839
20-pro-life	pro-life	is pro-life	213	1633
21-pro-choice	abortion	supports abortion	209	1593
22-physics	physics	is about physics	10360	93810
23-computer science	computers	is related to computer science	10441	93947
24-statistics	statistics	is about statistics	9286	86874
25-math	math	is about math research	8898	85118
26-grammar	ungrammatical	is ungrammatical	834	2217
27-grammar	grammatical	is grammatical	826	2236
28-sexis	sexist	is offensive to women	209	1641
29-sexis	feminism	supports feminism	215	1710
30-news	world	is about world news	5778	13023
31-sports	sports news	is about sports news	5674	12849
32-business	business	is related to business	5699	12913
33-tech	technology	is related to technology	5727	12927
34-bad	negative	contains a bad movie review	357	16889
35-good	good	thinks the movie is good	380	17497
36-quantity	quantity	asks for a quantity	1901	5144
37-location	location	asks about a location	1925	5236
38-person	person	asks about a person	1848	5014
39-entity	entity	asks about an entity	1896	5180
40-abbrevation	abbreviation	asks about an abbreviation	1839	5045
41-defin	definition	contains a definition	651	4508
42-environment	environmentalism	is against environmentalist	124	1117
43-environment	environmentalism	is environmentalist	119	1072
44-spam	spam	is a spam	360	2470
45-fact	facts	asks for factual information	704	11449
46-opinion	opinion	asks for an opinion	719	11709
47-math	science	is related to math and science	7514	53973
48-health	health	is related to health	7485	53986
49-computer	computers	related to computer or internet	7486	54256
50-sport	sports	is related to sports	7505	54718
51-entertainment	entertainment	is about entertainment	7461	53573
52-family	relationships	is about family and relationships	7438	54680
53-politic	politics	is related to politics or government	7410	53393

bioRxiv preprint doi: https://doi.org/10.1101/2025.08.12.669958; this version posted August 12, 2025. The copyright holder for this preprint (which was not certified by peer review) is the author/funder, who has granted bioRxiv a license to display the preprint in perpetuity. It is made available under aCC-BY 4.0 International license.

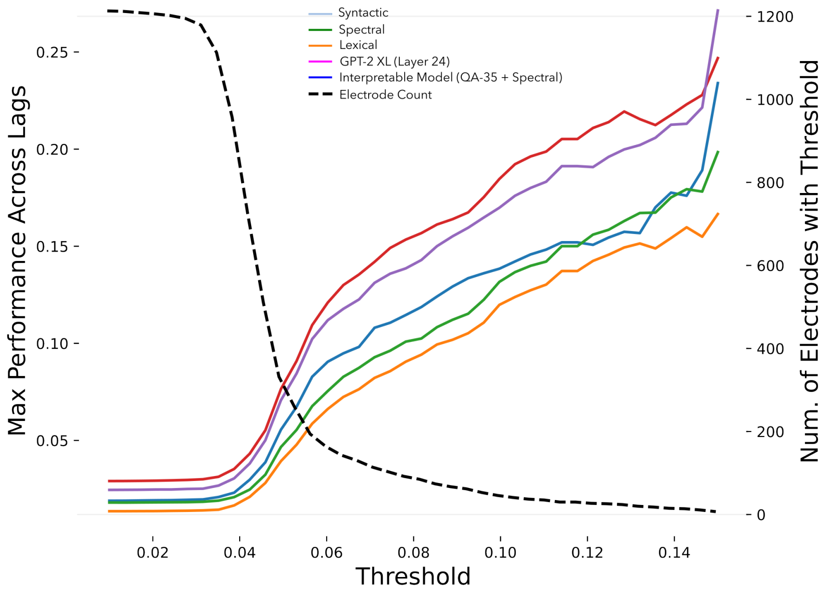

Figure S10: ECoG encoding model performance across test performance thresholds for selecting electrodes thresholds. The dashed line shows the number of electrodes that meet the threshold. Colored lines show the mean performance for each model from among the electrodes that meet the corresponding minimum threshold. We use a threshold of $r = 0.06$ for most of our analyses, approximately at the elbow of the dashed line.
